# Supplementary material for: Dynamical order and many-body correlations in zebrafish show that three is a crowd
Source: Nat Commun. 2024 Mar 22;15:2591. doi: 10.1038/s41467-024-46426-1 (PMC10959973; doi:10.1038/s41467-024-46426-1)
Supplement: Supplementary file 1 — Supplementary Information [file 41467_2024_46426_MOESM1_ESM.pdf]

Supplementary information
Dynamical Order and Many-Body Correlations in Zebrafish show that Three is a Crowd

Alexandra Zampetaki,[1,2] Yushi Yang,[3] Hartmut Löwen,[2] and C. Patrick Royall[3,4]

[1]*Institute for Applied Physics, TU Wien, A-1040 Wien, Austria*
[2]*Institut für Theoretische Physik II, Weiche Materie, Heinrich-Heine-Universität, 40225 Düsseldorf, Germany*
[3]*HH Wills Physics Laboratory, Tyndall Avenue, Bristol, BS8 1TL, UK*
[4]*Gulliver, UMR CNRS 7083, ESPCI Paris, Université PSL, 75005 Paris, France*

I. EXPERIMENTAL DETAILS

A. Typical Experimental Protocol

Here we describe our experiment protocol for recording small groups of fish. This recording procedure enables us to label and track the fish in each individual experiment. Operationally, we carry out the following tasks in a typical experiment.

1. We set up the observation room to have a fixed brightness ($\sim$ 25 LUX) and the water in the tank to have a fixed temperature ($\approx 25^\circ$ C).
2. We select three fish out of their living tanks without preference, and transfer them to the observation room.
3. We select two fish out of three for the first observation without preference, and place these two into the observation tank. We label the fish that was not chosen as fish C.
4. We start a recording timer, and then leave the observation room. The recordings automatically started 10 minutes later. The movements of the fish are recorded for 1 hour at a frame rate of 15fps. Therefore on each camera we capture 54000 frames in total in the MP4 video format.
5. We return to the observation room, and choose one fish in the observation tank without preference, and place it back to a temporary tank. This fish, being taken out from observation, is fish B, and the fish left for observation is fish A. We then place fish C into the observation tank to observe fish A and fish C. And then we repeat step 4.
6. We return to the observation room and place fish B into the observation tank, and then repeat step 4 to observe the triplet of fish A, B and C.
7. We return to the observation room, and repeat step 4 again to capture another 3-fish video.
8. We return to the observation room to place the fish back to their living tank, and use automatic script to process the videos.

To ensure our observed results are not biased, we repeated our observations 6 times, and concatenated all the results. The dates of the experiments are as follows: 2021-05-11, 2021-05-12, 2021-05-14, 2021-05-18, 2021-05-19, 2021-06-01.

B. Tracking the zebrafish

Here we carry out a 3D tracking protocol, which is explained in detail in Ref. [1]. Briefly, we first obtain 3D coordinates of the fish, and then link these locations into trajectories. This association process (to find the same fish in pictures taken from different cameras) can be carried out just by considering the multi-view geometry, as well as the refraction of water. We then followed the existing Lagrangian particle tracking algorithm to link these positions into trajectories [2, 3]. To tackle the 3D tracking task, we developed our own tracking method from scratch, which we discuss in our previous publication [1]. We also shared all the related source code on GitHub (https://github.com/yangyushi/FishPy).

While the linking process to connect fish coordinates between frames is not perfect and we encounter some breaking of trajectories, we are confident that our camera system and tracking code produces good results, because we tested them with simulated data. As described in Ref. [1], we simulated the trajectories of 50 Vicsek agents and rendered these agents in simulated conditions mimicking our experimental setup. Knowing the ground truth of the movement

of these agents, we re-constructed the movements with our algorithms. By comparing the ground truth with our reconstruction, we learned that the algorithm locates 45 fish out of 50. The missing fish would cause IDs to change and trajectories to break, but have a limited impact on the calculated order parameters and correlation functions.

COMPARISON BETWEEN SIMULATION AND EXPERIMENTAL RESULTS

We now present our two-dimensional (2D) simulation methods and the corresponding results for comparing with our experimental results shown in the main manuscript. As we will see, we find overall a good agreement of the simulated results with the experiment (Figs. 1, 3, 5) with some limitations coming from the simplified assumption of the tank shape being a 2D circle, instead of parabolic (Figs. 7). We also describe in more detail the procedure we use in order to calculate the three-body correlation and the Kirkwood approximation and present also the corresponding results for the milling and swarming states in experiment and simulations (Fig. 4).

II. SIMULATION METHODS

For our numerical simulations we use a perception model with the zebrafish interactions dominated by the repulsion-attraction between the fish. For completeness we include also weak alignment interactions, as well as some hydrodynamic interactions inspired by Ref. [4]. We produce for simplicity our main numerical results with a constant speed model, but we check also the effect of a variable fish speed and find it to be insignificant concerning the behaviour of our observables.

A. Constant speed model

Our main model is as follows. We assume that the fish move in 2D with a constant speed v_0 and can only change their velocity orientation, given by the angle ϕ_i (see also Fig. 2 (a), main text). Thus, the 2D overdamped equations of motion for fish i, positioned at $\mathbf{r}_i = (x_i, y_i)$ and heading along $\mathbf{e}_{i,v} = (\cos\phi_i, \sin\phi_i)$, read

$$\dot{x}_i = v_0 \cos\phi_i \quad \text{(Supplementary Eq.1)}$$

$$\dot{y}_i = v_0 \sin\phi_i \quad \text{(Supplementary Eq.2)}$$

$$\dot{\phi}_i = \frac{1}{v_0}\left(F^{(\text{wall})}_{i,\phi} + F^{(\text{int})}_{i,\phi} + \eta_{i,\phi}\right), \quad \text{(Supplementary Eq.3)}$$

where $\mathbf{F}^{(\text{wall})}_i$ stands for the interactions of fish i with the wall and $\mathbf{F}^{(\text{int})}_i$ denotes the total interactions of fish i with all the other fish. The subscript ϕ stands for the projection of the respective forces on the turning direction

$$\mathbf{e}_{i,\phi} = -\sin\phi_i \mathbf{e}_x + \cos\phi_i \mathbf{e}_y. \quad \text{(Supplementary Eq.4)}$$

The term $\eta_{i,\phi}$ stands for a zero-mean Gaussian noise with $\langle \eta_{i,\phi}(t)\eta_{j,\phi}(t+\tau)\rangle = 2D_\phi \delta_{ij}\delta(\tau)$.

In the following we consider reduced forces which have the dimension of length over time squared, allowing us to present all the quantities in units of L (fish body length) and $T = L/v_f$ (time). We assume that the fish are confined in a circular tank of radius $R = 66.7$ and model the wall avoidance by a soft repulsive potential resulting in a reduced force

$$\mathbf{F}^{(\text{wall})}_i = -C_w \frac{\exp[-\alpha_w (r_i - R)^4)]}{r_i}\mathbf{r}_i, \quad \text{(Supplementary Eq.5)}$$

with $r_i = \sqrt{x_i^2 + y_i^2}$, $C_w = 3$ the strength of the wall force and $\alpha_w = 8.1 \times 10^{-3}$. This yields the projected wall force

$$F^{(\text{wall})}_{i,\phi} = \mathbf{F}^{(\text{wall})}_i \cdot \mathbf{e}_{i,\phi} = C_w \frac{\exp[-\alpha_w (r_i - R)^4)]}{r_i}(x_i \sin\phi_i - y_i \cos\phi_i), \quad \text{(Supplementary Eq.6)}$$

used in the equations of motion (Supplementary Eq.3).

The interactions between fish i and any other fish j are assumed to consist of repulsion-attraction $\mathbf{f}^{(\text{att})}_{ij}$, alignment $\mathbf{f}^{(\text{al})}_{ij}$ and hydrodynamic interactions $\mathbf{f}^{(\text{hyd})}_{ij}$, so that the total interaction force acting on fish i reads

$$\mathbf{F}^{(\text{int})}_i = \sum_{j\neq i}\left(\mathbf{f}^{(\text{att})}_{ij} + \mathbf{f}^{(\text{al})}_{ij}\right) + \mathbf{f}^{(\text{hyd})}_{ij}. \quad \text{(Supplementary Eq.7)}$$

More specifically we assume that the repulsion-attraction interaction which is dominant in zebrafish [5, 6] is given by Morse interactions of the form

$$\mathbf{f}_{ij}^{(\mathrm{att})} = -\left(C_R \exp\left[-\frac{r_{ij}}{l_R}\right] - C_A \exp\left[-\frac{r_{ij}}{l_A}\right]\right) G(\theta_{ij}) \frac{\mathbf{r}_{ij}}{r_{ij}} \qquad \text{(Supplementary Eq.8)}$$

Here $\mathbf{r}_{ij} = \mathbf{r}_i - \mathbf{r}_j$, $r_{ij} = |\mathbf{r}_{ij}|$ is the distance between the fish i, j and $\theta_{ij} = \psi_{ji} - \phi_i$ denotes the perception angle in which fish i sees fish j (see Fig. 2 (a), main text for the definition of θ_{ij} and ψ_{ji}). The parameters C_R, C_A determine the strength of repulsion and attraction, while l_R, l_A determine their range. The values used in our simulations are given in table I. Among others, these values provide an equilibrium distance $r_{ij}^{(\mathrm{eq})} = 1$ equal to the average body length of the fish. The function $G(\theta_{ij})$ accounts for the anisotropic visual perception between the fish i and j, similarly to [4], and is here assumed to be given by

$$G(\theta_{ij}) = 1 + \epsilon \cos\theta_{ij}, \qquad \text{(Supplementary Eq.9)}$$

with $\epsilon = 0.3$ the perception parameter. The projected attraction interaction then reads

$$f_{ij,\phi}^{(\mathrm{att})} = \mathbf{f}_{ij}^{(\mathrm{att})} \cdot \mathbf{e}_{i,\phi} = -\left(C_R \exp\left[-\frac{r_{ij}}{l_R}\right] - C_A \exp\left[-\frac{r_{ij}}{l_A}\right]\right) G(\theta_{ij}) \sin\theta_{ij}. \qquad \text{(Supplementary Eq.10)}$$

In a similar way the alignment interaction is assumed to be

$$\mathbf{f}_{ij}^{(\mathrm{al})} = C_L \exp\left[-\frac{r_{ij}}{l_L}\right] G(\theta_{ij}) \left(\mathbf{e}_{i,v} - \mathbf{e}_{j,v}\right), \qquad \text{(Supplementary Eq.11)}$$

yielding its projection in the turning direction

$$f_{ij,\phi}^{(\mathrm{al})} = \mathbf{f}_{ij}^{(\mathrm{al})} \cdot \mathbf{e}_{i,\phi} = C_L \exp\left[-\frac{r_{ij}}{l_L}\right] G(\theta_{ij}) \sin\left(\phi_j - \phi_i\right) \qquad \text{(Supplementary Eq.12)}$$

with $C_L = 0.075$ and $l_L = 1.33$. Evidently, the alignment interactions are very suppressed for zebrafish as pointed out also in other studies.

We have observed that for a system to exhibit a milling state for $N = 3$ particles (swimmers) under the above assumptions of attraction-dominated behavioural interactions, hydrodynamic interactions are essential. In order to account for the hydrodynamic interactions we follow the approach of [4], considering only the far-field dipolar flow generated by the swimmers. Under this approach, the hydrodynamic interactions result in a drift term

$$\mathbf{U}_i = \sum_{j\neq i} \mathbf{u}_{ij} = \sum_{j\neq i} \frac{I_f}{\pi r_{ij}^2} \left[\mathbf{e}_j^{\psi} \sin\theta_{ji} + \mathbf{e}_j^{r} \cos\theta_{ji}\right]. \qquad \text{(Supplementary Eq.13)}$$

In the above, I_f characterizes the swimmer dipole intensity, $\theta_{ji} = \psi_{ij} - \phi_j$ with $\psi_{ij} = -\pi + \psi_{ji}$ and the unit vectors $\mathbf{e}_j^{\psi}, \mathbf{e}_j^{r}$ read

$$\begin{aligned} \mathbf{e}_j^{\psi} &= \sin\psi_{ji}\mathbf{e}_x - \cos\psi_{ji}\mathbf{e}_y \\ \mathbf{e}_j^{r} &= -\cos\psi_{ji}\mathbf{e}_x - \sin\psi_{ji}\mathbf{e}_y. \end{aligned} \qquad \text{(Supplementary Eq.14)}$$

Since in our case we assume that only the velocity orientation can change, the effect of the drift term $\mathbf{U}$ is captured by its projection along the unit vector $\mathbf{e}_{i,\phi}$. Thus, we assume that the hydrodynamic interaction of the swimmer i with the swimmer j projected in the turning direction can be approximated by

$$f_{ij,\phi}^{(\mathrm{hyd})} = \mathbf{u}_{ij} \cdot \mathbf{e}_{i,\phi} = \frac{C_H}{r_{ij}^2} \sin\left(2\psi_{ji} - \phi_i - \phi_j\right), \qquad \text{(Supplementary Eq.15)}$$

where C_H is a parameter controlling the strength of the hydrodynamic interaction and here chosen to have the value $C_H = 0.25$. The total turning interactions then read

$$F_{i,\phi}^{(\mathrm{int})} = \sum_{j\neq i} \left(f_{ij,\phi}^{(\mathrm{att})} + f_{ij,\phi}^{(\mathrm{al})}\right) + f_{ij,\phi}^{(\mathrm{hyd})}. \qquad \text{(Supplementary Eq.16)}$$

Constant Speed Simulation Parameters

Self-propulsion speed	v_0	$[L/T]$	1
Rotational diffusion	D_ϕ	$[L^2/T^3]$	0.015
Wall force strength	C_w	$[L/T^2]$	3
Wall force parameter	α_w	$[1/L^4]$	8.1×10^{-3}
Repulsion strength	C_R	$[L/T^2]$	11.01
Attraction strength	C_A	$[L/T^2]$	3
Repulsion range	l_R	$[L]$	0.67
Attraction range	l_A	$[L]$	5
Alignment strength	C_L	$[L/T^2]$	0.075
Alignment range	l_L	$[L]$	1.33
Hydrodynamic interactions strength	C_H	$[L^3/T^2]$	0.25
Perception angle parameter	ϵ	[1]	0.3
Maximum rotation angle	$\Delta\phi_{max}$	[1]	$\pi/6$
Tank radius	R	$[L]$	66.7
Time step	Δt	$[T]$	0.067

Supplementary Table I. Summary of the simulation parameters for our constant speed model. The third column shows the units of the parameters in terms of arbitrary length L and time T units. Here we choose as a length unit the average body length of the zebrafish $L = 30$ mm and a time unit so that the average speed of zebrafish v_0 is one, i.e. $T = L/v_0 = 30$ mm /100(mms^{-1})=0.3 s. In these units the parameter values used to obtain the results of Figs. 1, 2, 3, 4 and 5 are shown in the fourth column.

The rest of the parameters used in simulations read $D_\phi = 0.015$ and $v_0 = 1$. In order to avoid arbitrarily large changes in the fish orientation we introduce a maximum value of rotation $\Delta\phi_{\max} = \frac{\pi}{6}$, similarly to Ref. [7]. In all cases the particles are initialized in random positions inside the circular tank with uniformly random orientations $\phi_i \in [-\pi, \pi]$. We integrate the equations of motion (Eqs. (Supplementary Eq.1)-(Supplementary Eq.3)) with a time step $\Delta t = 0.067$ for 10000 steps and average over 50 different realizations, so that we emulate better the experimental situation. We summarize all the parameters used in our simulations with constant speed at Table I. We remark that we use this single set of parameters to obtain all our results, unless stated otherwise.

B. Variable speed

We also use a more involved model, allowing for variations in the fish speed and investigate this effect upon the order parameters we measure. There are several studies which employ such speed variations in the modelling of fish behavior [8–11]. Here we follow the procedure described in [11]. In particular, we change our equations of motion (Supplementary Eq.3) to include also speed changes due to the wall force $\mathbf{F}_i^{(\text{wall})}$, the interactions with other fish $\mathbf{F}_i^{(\text{int})}$ and noise $\eta_{i,v}$. These then take the form

$$\dot{x}_i = v_i \cos\phi_i \quad \text{(Supplementary Eq.17)}$$

$$\dot{y}_i = v_i \sin\phi_i \quad \text{(Supplementary Eq.18)}$$

$$\dot{\phi}_i = \frac{1}{v_i + \alpha}\left(F_{i,\phi}^{(\text{wall})} + F_{i,\phi}^{(\text{int})} + \eta_{i,\phi}\right) \quad \text{(Supplementary Eq.19)}$$

$$\dot{v}_i = \beta\,(v_0 - v_i) + F_{i,v}^{(\text{wall})} + F_{i,v}^{(\text{int})} + \eta_{i,v}. \quad \text{(Supplementary Eq.20)}$$

The pre-factor $\frac{1}{v_i+\alpha}$ in the third equation captures the expectation that fish with larger speeds should turn more slowly in response to a certain force. Note that in the case of constant speed this just leads to a rescaling of all the forces and the noise strength with the self-propulsion velocity v_0. The friction coefficient α here has the value $\alpha = 0.2$.

Regarding the speed update, the first term $\beta\,(v_0 - v_i)$ stands for the tendency of the fish to maintain a preferred speed v_0 with a relaxation coefficient $\beta = 10$. The next term $F_{i,v}^{(\text{wall})}$ is the wall force along the heading direction, i.e.

$$F_{i,v}^{(\text{wall})} = \mathbf{F}_i^{(\text{wall})} \cdot \mathbf{e}_{i,v} = -C_w \frac{\exp[-\alpha_w (r_i - R)^4]}{r_i}(x_i \cos\phi_i + y_i \sin\phi_i). \quad \text{(Supplementary Eq.21)}$$

For the interactions $\mathbf{F}^{(\text{int})}$ we here assume a very similar expression to Supplementary Eq.7, but because these also affect the speed update we find it necessary to restrict the summation of the social forces $\mathbf{f}_{ij}^{(att)}$ and $\mathbf{f}_{ij}^{(al)}$ within the Voronoi neighbourhood $\mathcal{N}_i$ of agent i [11], i.e.

Variable Speed Simulation Parameters

Self-propulsion speed	v_0	$[L/T]$	1
Turning friction	α	$[L/T]$	0.2
Relaxation coefficient	β	$[1/T]$	30
Velocity diffusion	D_v	$[L^2/T^3]$	2.4
Rotational diffusion	D_ϕ	$[L^2/T^3]$	0.06
Wall force strength	C_w	$[L/T^2]$	12
Wall force parameter	α_w	$[1/L^4]$	8.1×10^{-3}
Repulsion strength	C_R	$[L/T^2]$	44.04
Attraction strength	C_A	$[L/T^2]$	12
Repulsion range	l_R	$[L]$	0.67
Attraction range	l_A	$[L]$	5
Alignment strength	C_L	$[L/T^2]$	0.3
Alignment range	l_L	$[L]$	1.33
Hydrodynamic interactions strength	C_H	$[L^3/T^2]$	1
Perception angle parameter	ϵ	[1]	0.5
Maximum rotation angle	$\Delta\phi_{max}$	[1]	$\pi/6$
Tank radius	R	$[L]$	66.7
Time step	Δt	$[T]$	0.033

Supplementary Table II. Summary of the simulation parameters for our variable speed model. The third column shows the units of the parameters in terms of arbitrary length L and time T units. Here we choose as a length unit the average body length of the zebrafish $L = 30$ mm and a time unit so that the average speed of zebrafish v_0 is one, i.e. $T = L/v_0 = 30$ mm/100(mms^{-1}) = 0.3s. In these units the parameter values used to obtain the results of Fig. 6 are shown in the fourth column.

$$\mathbf{F}_i^{(\text{int})} = \frac{1}{|\mathcal{N}_i|} \sum_{j \in \mathcal{N}_i} \left(\mathbf{f}_{ij}^{(\text{att})} + \mathbf{f}_{ij}^{(\text{al})} \right) + \sum_{j \neq i} \mathbf{f}_{ij}^{(\text{hyd})}. \qquad \text{(Supplementary Eq.22)}$$

Their projection $F_{i,\phi}^{(\text{int})} = \mathbf{F}_i^{(\text{int})} \cdot \mathbf{e}_{i,\phi}$ affects the velocity orientation, as in the case of the constant speed model, whereas $F_{i,v}^{(\text{int})} = \mathbf{F}_i^{(\text{int})} \cdot \mathbf{e}_{i,v}$ causes changes in the agent's speed. The contributions of the different interactions in such speed variations are given by

$$f_{ij,v}^{(\text{att})} = \mathbf{f}_{ij}^{(\text{att})} \cdot \mathbf{e}_{i,v} = -\left(C_R \exp\left[-\frac{r_{ij}}{l_R} \right] - C_A \exp\left[-\frac{r_{ij}}{l_A} \right] \right) G(\theta_{ij}) \cos\theta_{ij}, \qquad \text{(Supplementary Eq.23)}$$

$$f_{ij,v}^{(al)} = \mathbf{f}_{ij}^{(\text{al})} \cdot \mathbf{e}_{i,v} = C_L \exp\left[-\frac{r_{ij}}{l_L} \right] G(\theta_{ij}) \left(\cos\left(\phi_j - \phi_i\right) - 1 \right) \qquad \text{(Supplementary Eq.24)}$$

and

$$f_{ij,v}^{(\text{hyd})} = \mathbf{u}_{ij} \cdot \mathbf{e}_{i,v} = \frac{C_H}{r_{ij}^2} \cos\left(2\psi_{ji} - \phi_i - \phi_j\right). \qquad \text{(Supplementary Eq.25)}$$

The random fluctuations of the speed are represented by the term zero-mean Gaussian noise $\eta_{i,v}$ with

$$\langle \eta_{i,v}(t) \eta_{j,v}(t+\tau) \rangle = 2 D_v \delta_{ij} \delta(\tau) \qquad \text{(Supplementary Eq.26)}$$

and $D_v = 2.4$. The values of the parameters producing qualitatively similar results to the experiment for this model are presented at the Table II.

In all cases, we start our simulations from uniform random positions in the central region of the tank with random orientations and uniformly random speeds in the interval $[v_0 - 0.05, v_0 + 0.05]$. As in the case of our constant speed model, we repeat them for 10000 steps and average over 50 realisations.

III. SIMULATION RESULTS

In this section we present some of our simulation results corresponding to the experimental data described in the main text. In general we have observed that despite the plethora of models describing the behavior of fish in literature e.g. [4, 7, 12], it is challenging to find a single model that captures correctly both the intermittent dynamics of different numbers of fish and their two and three-body correlations in the different states. This is particularly hard in the case of zebrafish whose behavioral interactions are dominated by their attraction, which e.g. as a rule does not allow for a strong exhibition of the milling state found regularly in experiments. As an example the well-known zonal model [7] predicts a transition to milling for sufficiently large range of alignment interaction, which is evidently not the case for zebrafish.

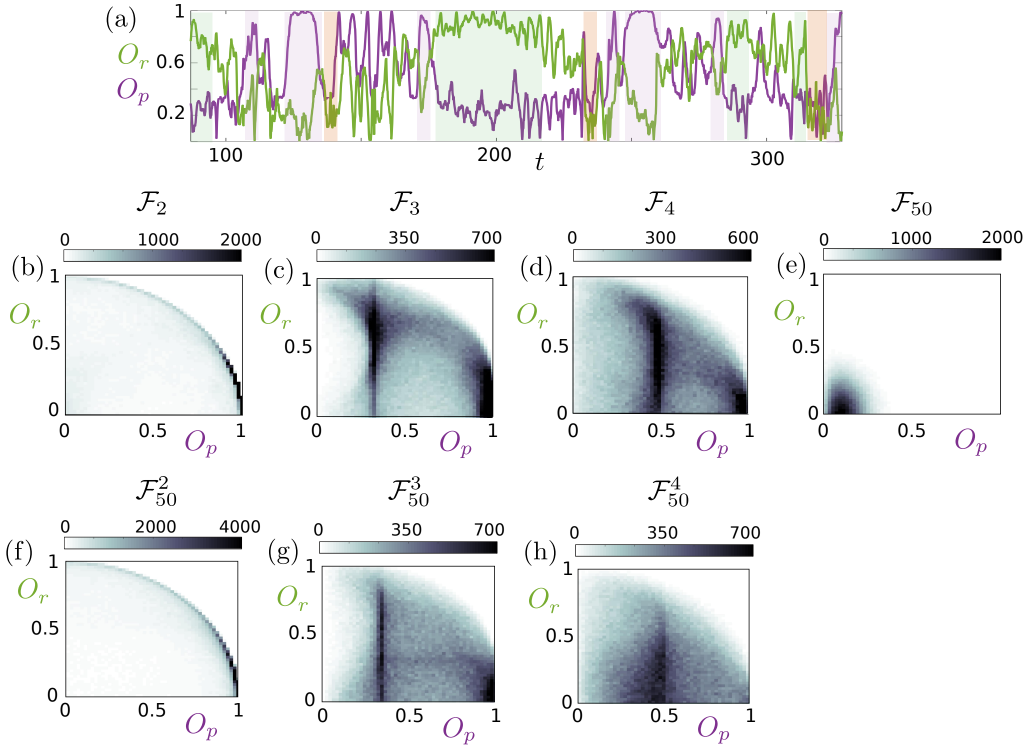

Supplementary Fig. 1. Three fish exhibit schooling, milling and swarming states in simulations. (a) Time evolution of the rotational and polarisation order parameters O_r and O_p respectively, for a 3-fish simulation trajectory. The times where the different states are found are marked with a different colour: purple for schooling, green for milling and orange for swarming, similarly to the discussion in the main text (see Fig. 3). (b)-(e) Density plots of O_r versus O_p for (b) $N = 2$ fish (c) $N = 3$ fish (d) $N = 4$ fish (e) $N = 50$ fish, pointing to the occurrence of the different states in simulations. (f)-(h) Density plots of O_r versus O_p for small local groups of $N = 50$ fish, consisting of n nearest neighbouring fish ($\mathcal{F}_{50}^n$):(f) $n = 2$ ($\mathcal{F}_{50}^2$),(g) $n = 3$ ($\mathcal{F}_{50}^3$) and (h) $n = 4$ ($\mathcal{F}_{50}^4$). The simulation parameters used are mentioned in the method section.

Within our 2D model, taking into account also some form of hydrodynamic interactions, as described above, we find an intermittent behaviour for $N = 3$ fish ($\mathcal{F}_3$) despite the presence of strong attraction [Fig. 1 (a)]. This behaviour is similar to that found in experiments (Fig. 3, main text) and shows that 3 fish can exhibit schooling, milling and swarming states also in simulations. Even more, the different group sizes of fish $\mathcal{F}_{2,3,4,50}$ in simulations [Fig. 1 (b)-(e)] behave in a qualitatively similar way to those in the experiments with the weight of the swarming state increasing for increasing number of fish. We note here that this transition to a swarming state is gradual as indicated by the O_p, O_r density plots for $\mathcal{F}_{5,6,7,8,9,10}$ shown in Fig. 2. Furthermore, in the simulations we verify as well that local subgroups of larger groups of fish, $\mathcal{F}_{50}^{2,3,4}$, [Fig. 1 (f)-(h)] behave like the corresponding isolated groups of fish. The largest discrepancy between the simulations and experiment in this case is the very pronounced peak at certain values of O_p in the simulation results, which corresponds to the state with two fish being aligned and the third fish anti-aligned. As we discuss below, this can be attributed to the reduced 2D geometry of the simulations, compared to the 3D parabolic confinement of the experiments.

More detailed information on the system is provided, as discussed in the main text, by the two and three-body

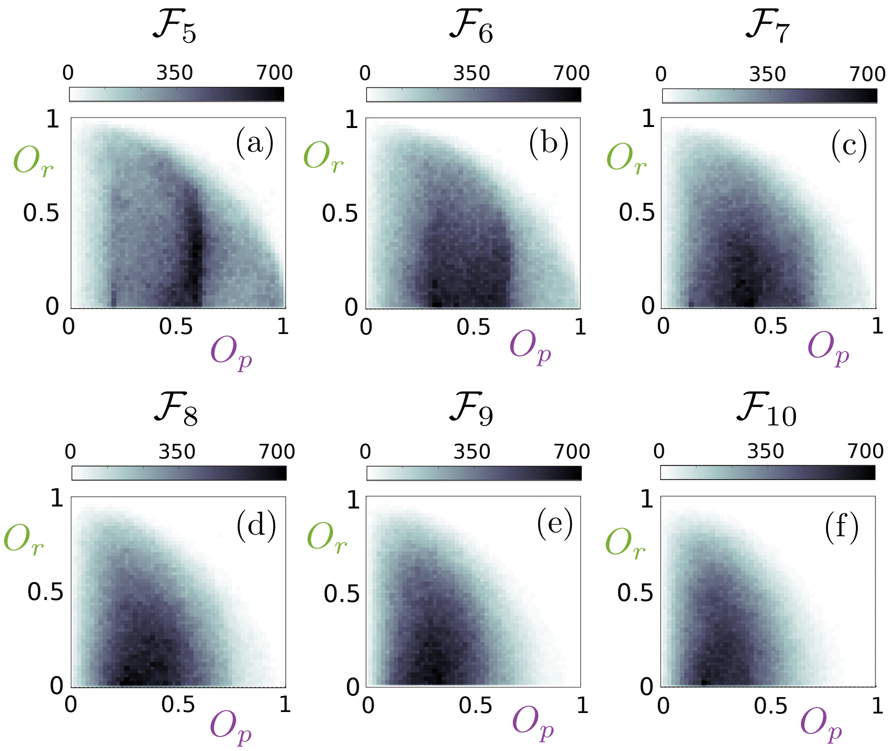

Supplementary Fig. 2. Density plots of O_r versus O_p from simulations of (a) $N = 5$ fish (b) $N = 6$ fish (c) $N = 7$ fish (d) $N = 8$ fish (e) $N = 9$ fish (f) $N = 10$ fish. They indicate that the transition to the swarming state for an increasing group size of fish is rather a crossover and not an actual phase transition. The simulation parameters used are mentioned in the method section.

correlations. Considering the two-body correlations in the reference frame of one fish $g_2(x', y')$ we observe that the simulations generally capture the difference between the spatial correlations in the different states. In particular, whereas in the schooling state there are more prominent front-back peaks, in the milling state one can observe peaks on the side [Fig. 3 (a1),(a2),(b1),(b2)] as in the experiment (Fig. 4, main text). We find a discrepancy in the $g_2(x', y')$ for $\mathcal{F}_3$ in the swarming state [Fig. 3 (a3)], as well as in the $g_2(x', y')$ for $\mathcal{F}^3_{50}$ in the schooling state [Fig. 3 (b1)]. Also in simulations there is a high population of the peak directly behind the reference fish, which is absent in experiments. Such discrepancies might be again connected with the 2D confinement of the simulations or the incomplete account of the anisotropic vision of the fish and their avoidance interactions. Regarding the three-fish bond angle $\theta_r^{(3)}$ distribution [Fig. 3 (c)], we observe that it seems to show less variation between the different states and the number of particles than that in the experiments.It is generally characterized by a peak around 45°, similarly to the $\mathcal{F}^3_{50}$ case in experiments (Fig. 4 (c), main text). Overall we see that in the simulations the local groups $\mathcal{F}^3_{50}$ show more similarities in their correlations with the isolated groups $\mathcal{F}^3$ than that found in experiments.

The spatial three-body correlations $g_3(\tilde{x}_3, \tilde{y}_3)$ in the milling and swarming look quite similar [Fig. 4 (a1),(b1)] and show that there is an increased probability to find the third fish on the side between the two other fish. This behaviour is very well captured by the Kirkwood superposition approximation $g_3^K(\tilde{x}_3, \tilde{y}_3)$ [Fig. 4 (a2),(b2)] and qualitatively also captured by simulations [Fig. 4 (c1)-(d1)].

For the simulation results of g_3 for the schooling state we have performed the same analysis as for the experiments, distinguishing between the orientations of the reference fish (Fig. 5). Similarly to the experiments (Fig. 5, main text) we see that when the reference fish swim head-to-tail there is increased probability of the third fish to lie between them, whereas when they swim side-by-side the third fish swims most probably in the front or the back of the reference fish [Fig. 5 (b1)-(b3)]. This effect is suppressed for local groups of three fish [Fig. 5 (c1)-(c3)], but still not to the same degree as in experiment. Additionally, unlike the experiment there is no strong peak in the middle between the head-to-tail swimming fish in the simulations' $\mathcal{F}_3$ case and the overall behaviour is well captured by the Kirkwood superposition approximation [Fig. 5 (a1)-(a3)]. This could imply the existence of some three-body interactions in the experimental case of three fish.

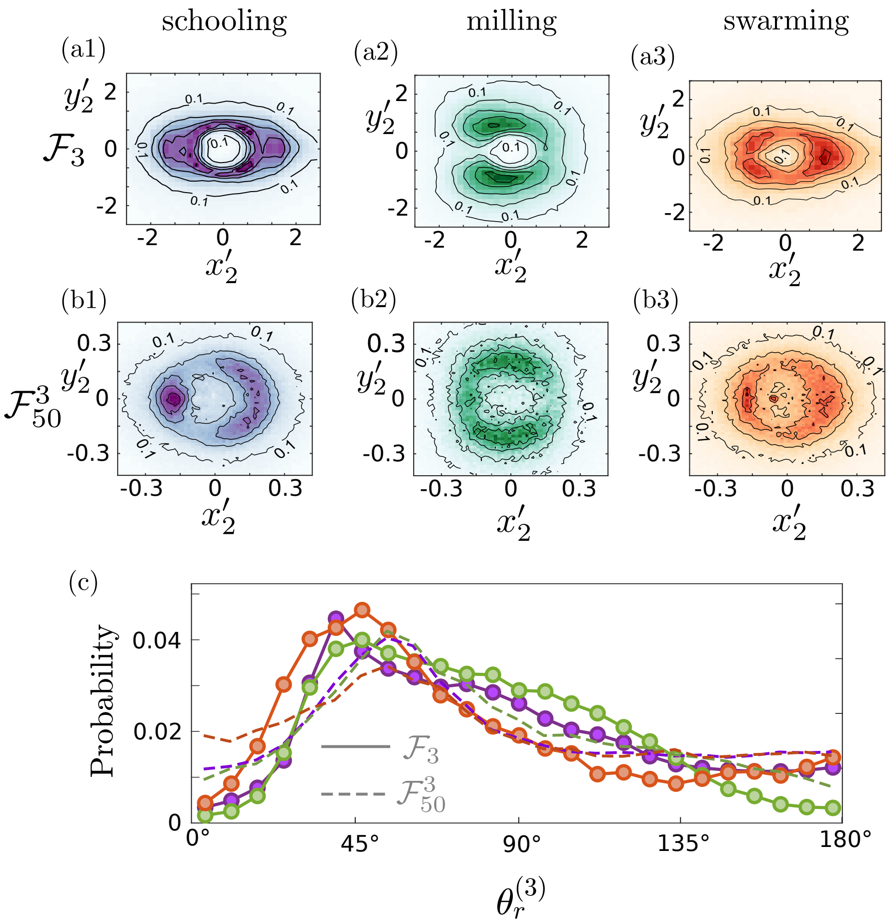

Supplementary Fig. 3. Pair correlations g_2 in the reference frame of one of the fish for the different states as found in simulations of (a1)-(a3): $N = 3$ fish ($\mathcal{F}_3$) and (b1)-(b3) local groups of $n = 3$ NN fish in the case of $N = 50$ fish ($\mathcal{F}^3_{50}$). We distinguish between the different states as follows (a1,b1) schooling, (a2,b2) milling and (a3,b3) swarming state. Here for clarity we have normalized the correlation distributions by the maximum value, so that the colorbar goes form 0 (white) to 1 (maximum saturation). The contour lines correspond to 0.1, 0.3, 0.5, 0.7 and 0.9. (c) Probability distribution of the three-fish bond angle $\theta_r^{(3)}$ of $N = 3$ fish in the milling (green line with circles), the swarming (orange line with circles) and the schooling (purple line with circles) state. The dashed lines show the corresponding results for $\mathcal{F}^3_{50}$. The simulation parameters used are mentioned in the method section.

A. Effects of variable speed

We now discuss the effect of a speed variation, taken into account within our variable speed model discussed in Section II. B. The obtained speed distributions [Fig. 6 (b)] are quite similar to those obtained in the experiments. [Fig. 6 (a)], especially concerning the fact that while for the $\mathcal{F}_3$ system the distributions are peaked around the preferred speed v_0, for the $\mathcal{F}_{50}$ system they are peaked at lower values. It seems therefore that both in experiments and in simulations the fish adjust their speed collectively so that they can swim more easily in a crowded environment. A similar effect has been found in [11] and has been attributed to a topological transition from an all-to-all coupling for $N \leq 3$ to a distributed spatial interaction network for $N > 3$. The main difference between the experimental and simulation distributions is that the former have a rather asymmetric profile, steeper for lower speeds whereas the latter have symmetric distributions. This could be attributed to an inhomogeneous initial distribution of speeds in the fish or to the presence of different behavioural modes between which fish alternate during swimming [13].

The variations of speed seem he main observables of this study significantly rendering them similar to results from both the experiments constant speed model. We can see the similarity between the two models, for instance, in the order parameter density plots [Figs. 6 (c),(d) vs. Figs. 1 (c),(e)], in the pair correlations g_2 [Figs. 6 (e),(f) vs. Figs. 3 (a1),(a2)], as well as in the three body correlations g_3 [Figs. 6 (g),(h) vs. Figs. 5 (b1), 4 (c1)].

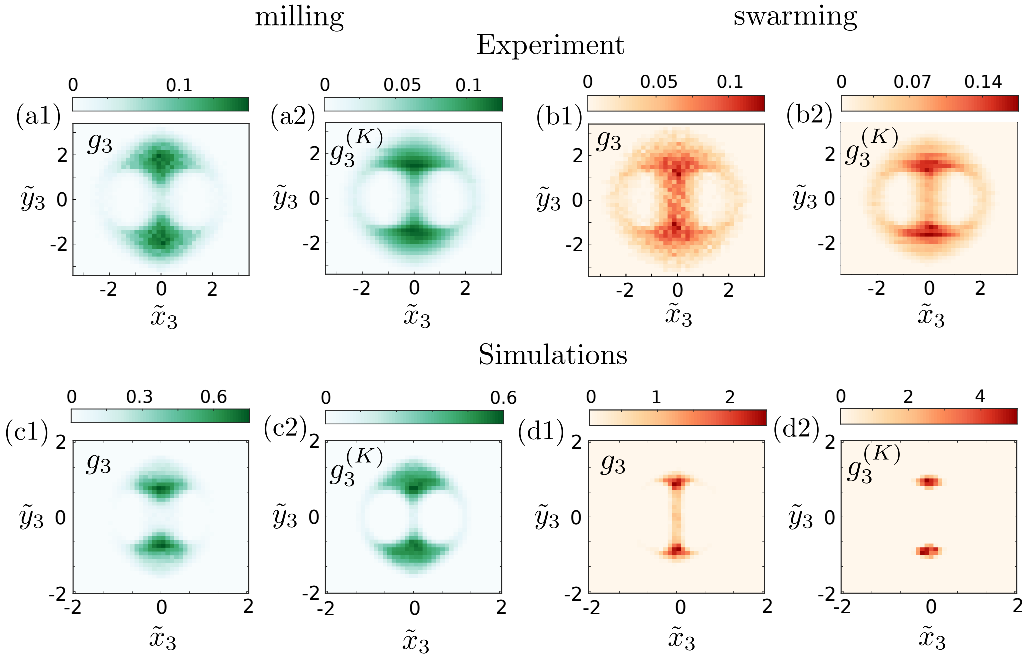

Supplementary Fig. 4. Analysis of three-body correlations of $N = 3$ fish ($\mathcal{F}_3$) in the milling state (a,c) and the swarming state (b,c) as found in experiment (first row) and in simulations (second row). Panels (a1,b1,c1,d1) show the full three fish correlation g_3 for $\mathcal{F}_3$, whereas panels (a2,b2,c2,d2) show the Kirkwood superposition approximation of the three fish correlation $g_3^{(K)}$ for $\mathcal{F}_3$.

B. Effects of geometry and interactions

In order to gain some understanding of the effects of geometry and the interactions on the dynamical behaviour of the three fish system ($\mathcal{F}_3$)we compare our results to that of an "ideal gas" system where there are no interactions between the three particles and their velocities are sampled from a uniformly random distribution. We examine three different confining geometries (tank shapes): a 2D circle of radius $R = 600$ [Fig. 7 (a)], a 3D sphere of radius $R = 600$ [Fig. 7 (b)] and a thin spherical segment ($-\frac{1}{2} < \theta < \frac{1}{2}$) which can be perceived as quasi-2D [Fig. 7 (c)]. We compare the results with an estimation of the non-interacting case from our experimental results for three fish [Fig. 7 (d)]. In order to ignore the fish interactions and the subsequent correlations, we choose random triplets out of the whole dataset of our three fish [$\mathcal{F}_3$] experiment, so that we can focus on the effect of confinement.

We find that the dimensionality of the confinement significantly affects the order parameter O_r-O_p distributions. In particular for the 2D case [Fig. 7 (a)] there is a prominent peak at $O_p \approx 0.33$ which corresponds to the case where two fish are aligned and one fish is anti-aligned. The same peak appears also in our 2D simulations [Fig. 1 (b)] and can be thus attributed to the tank geometry. The situation is very different for the 3D case where the distribution is much flatter and mostly concentrated in around the central values of $O_r, O_p \approx 0.5$. As should be expected, the spherical segment case lies somewhere between the 2D and the 3D cases [Fig. 1 (c)]. A comparison of these distributions to that approximated by our experimental values reveals that the parabolic confinement has effects similar to the quasi-2D case of the spherical segment [Fig. 1 (c)]. This fact explains some of the deviations of our experimental results from our 2D simulations, that is to say the difference of the confining geometry.

C. Three-body correlations and Kirkwood approximation

For three particles the radial three-body correlations can be characterized by the distribution $g_3(r_{12}, r_{23}, r_{31})$ with r_{ij} denoting the distance between the particles i and j. Since this is a 3D object, in order to visualize it in 2D we use its 2D projection $g_3(\tilde{x}_3, \tilde{y}_3)$. This is calculated as follows. We restrict the distance of one pair of particles, without loss of generality r_{12}, within a small window of width $W \approx 0.5L$ around its most probable value $r_{\max}$, i.e. the peak of $g_2(r_{12})$. Then, assuming the x-axis to coincide with the orientation of $\mathbf{r}_{12}$, and the origin with the midpoint $\mathbf{r}_O = (\mathbf{r}_1 + \mathbf{r}_2)/2$, we find the 2D distribution of the third fish position $g_3(\tilde{x}_3, \tilde{y}_3)$. Here it is implied that $\tilde{x}_3 = x_3 - x_O$

schooling

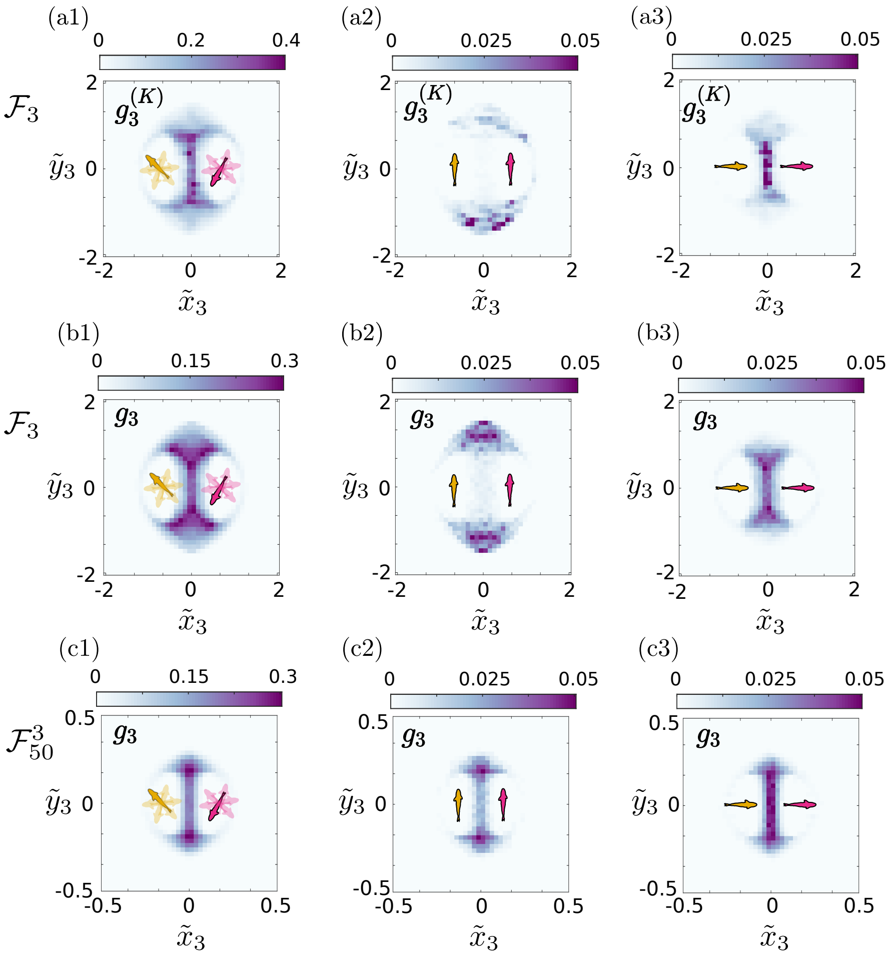

Supplementary Fig. 5. Analysis of three-body correlations of (a1)-(b3) $N = 3$ fish ($\mathcal{F}_3$) and (c1)-(c3) local group of $n = 3$ fish ($\mathcal{F}_{50}^3$) fish in the schooling state as found in simulations. The first row (a1-a3) shows the Kirkwood superposition approximation of the three fish correlation $g_3^{(K)}$ for $\mathcal{F}_3$. The second row (b1,b2,b3) shows the full three fish correlation g_3 for $\mathcal{F}_3$ and the last row (c1,c2,c3) shows g_3 for $\mathcal{F}_{50}^3$. In (a1,b1,c1) we present only the spatial three-body correlations $g_3^{(K)}$, g_3, without resolving the orientation of the two reference fish. In (a2,b2,c2) we present the three-body correlations $g_3^{(K)}$, g_3 for the reference fish aligned side-by-side whereas in (a3,b3,c3) we show $g_3^{(K)}$, g_3 for the reference fish aligned head-to-tail.

and $\tilde{y}_3 = y_3 - y_O$. For the bond angle $\theta_r^{(3)}$ we also select the values of r_{13} within a width W around $r_{\max}$ and compute the bond angle as $\theta_r^{(3)} = \cos^{-1}\left(\frac{\mathbf{r}_{12}\cdot\mathbf{r}_{13}}{r_{12}\ r_{13}}\right)$.

Meanwhile, the Kirkwood approximation reads

$$g_3^{(K)}(r_{12}, r_{23}, r_{31}) = g_2(r_{12})g_2(r_{23})g_2(r_{31}) \approx g_3(r_{12}, r_{23}, r_{31}). \qquad \text{(Supplementary Eq.27)}$$

For its 2D visualization we use instead the expression

$$g_3^{(K)}(\tilde{x}_3, \tilde{y}_3) = \int_{r_{\max}-W}^{r_{\max}+W} dr\ g_2(r) g_2\left(\sqrt{\left(\tilde{x}_3 - \frac{r}{2}\right)^2 + \tilde{y}_3^2}\right) g_2\left(\sqrt{\left(\tilde{x}_3 + \frac{r}{2}\right)^2 + \tilde{y}_3^2}\right), \qquad \text{(Supplementary Eq.28)}$$

which is the equivalent for the approach to compute the 2D visualization of g_3, namely $g_3(\tilde{x}_3, \tilde{y}_3)$. More information about the calculation and visualization of three-body correlations can be found at Ref. [14].

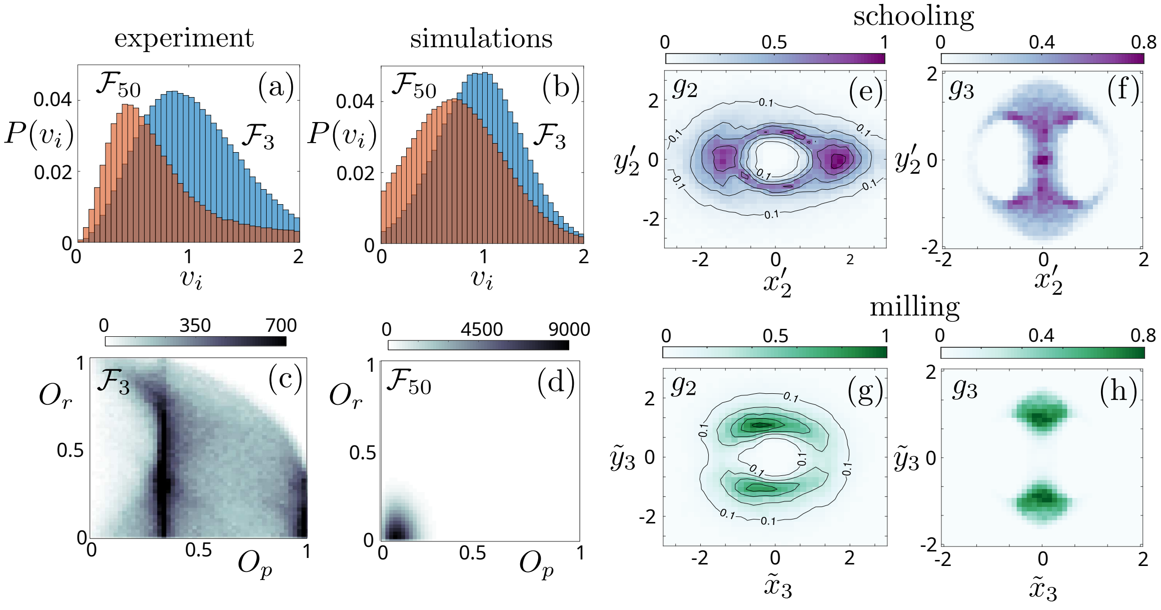

Supplementary Fig. 6. Simulation results of the variable speed model. (a,b) Speed distributions for the three fish system $\mathcal{F}_3$ (blue) and the 50 fish system $\mathcal{F}_{50}$ (orange) for (a) experiments and (b) simulations. (c,d) Density plots of O_r versus O_p for simulations of (c) $N = 3$ fish $\mathcal{F}_3$ and (d) $N = 50$ fish $\mathcal{F}_{50}$. (e,f) Pair correlations g_2 in the reference frame of one of the fish for the (e) the schooling state and (f) the milling state as found in variable speed simulations of $N = 3$ fish. (g,h) Full three fish correlation g_3 for (g) the schooling state and (h) the milling state derived for simulations of $N = 3$ fish.

Supplementary References

---

[1] Yang, Y. *et al.* Dominating lengthscales of zebrafish collective behaviour. *PLoS Computational Biology* **18**, 1–14 (2022).

[2] Ouellette, N. T., Xu, H. & Bodenschatz, E. A quantitative study of three-dimensional Lagrangian particle tracking algorithms. *Experiments in Fluids* **40**, 301–313 (2005).

[3] Xu, H. Tracking lagrangian trajectories in position–velocity space. *Measurement Science and Technology* **19**, 075105 (2008).

[4] Filella, A., Nadal, F. m. c., Sire, C., Kanso, E. & Eloy, C. Model of collective fish behavior with hydrodynamic interactions. *Phys. Rev. Lett.* **120**, 198101 (2018).

[5] Calovi, D. S. *et al.* Disentangling and modeling interactions in fish with burst-and-coast swimming reveal distinct alignment and attraction behaviors. *PLOS Computational Biology* **14**, 1–28 (2018).

[6] Escobedo, R. *et al.* A data-driven method for reconstructing and modelling social interactions in moving animal groups. *Trans. R. Soc. B* **375**, 20190380 (2020).

[7] Couzin, I. D., Krause, J., James, R., Ruxton, G. D. & Franks, N. R. Collective memory and spatial sorting in animal groups. *Journal of Theoretical Biology* **218**, 1–11 (2002).

[8] Herbert-Read, J. E. *et al.* How predation shapes the social interaction rules of shoaling fish. *Proc. R. Soc. B.* **284**, 20171126 (2017).

[9] Jolles, J. W., Boogert, N. J., Sridhar, V. H., Couzin, I. D. & Manica, A. Consistent individual differences drive collective behavior and group functioning of schooling fish. *Curr. Biol.* **27**, 2862–2868.e7 (2017).

[10] Jolles, J. W. *et al.* Group-level patterns emerge from individual speed as revealed by an extremely social robotic fish. *Biol. Lett.* **16**, 20200436 (2020).

[11] Klamser, P. P. *et al.* Impact of variable speed on collective movement of animal groups. *Frontiers in Physics* **9**, 715996 (2021). 2106.00959.

[12] Gautrais, J. *et al.* Deciphering interactions in moving animal groups. *PLoS Computational Biology* **8**, e1002678 (2012).

[13] Harpaz, R., r Tkacik, G. & Schneidman, E. Discrete modes of social information processing predict individual behavior of fish in a group. *Proceedings of the National Academy of Sciences* **114**, 10149–10154 (2017).

[14] Rüss, C., Zahn, K. & von Günberg, H.-H. Triplet correlations in two-dimensional colloidal model liquids. *Journal of Physics: Condensed Matter* **15**, S3509 (2003).

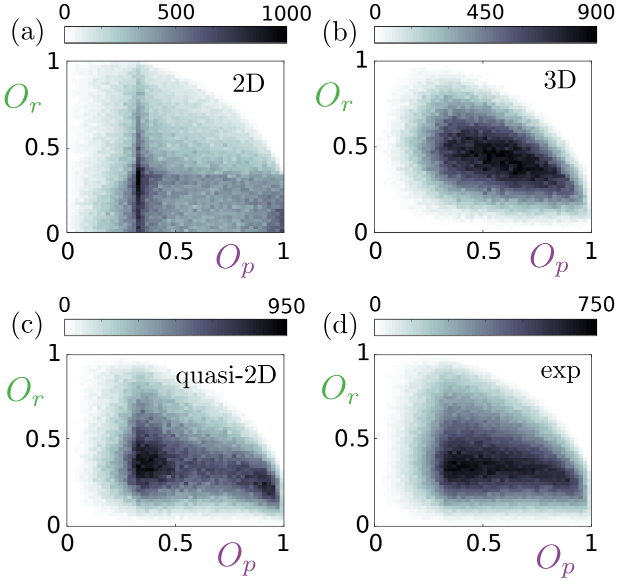

Supplementary Fig. 7. Density plots of O_r versus O_p in the ideal gas case $N = 3$ where there exist no interactions between the three particles (fish) for different confining geometries (tank shapes): (a) 2D circle, (b) 3D sphere and (c) quasi-2D spherical segment ($-\frac{1}{2} < \theta < \frac{1}{2}$). For comparison we present also in subfigure (d) an estimation from the experimental results, in which, in order to ignore the fish interactions and the subsequent correlations, we choose random triplets out of the whole dataset of our three fish ($\mathcal{F}_3$) experiment. The actual results for the latter are shown in Fig. 3 (f) in the main text.
