# Supplementary material for: Dynamical order and many-body correlations in zebrafish show that three is a crowd
Source: Nat Commun. 2024 Mar 22;15:2591. doi: 10.1038/s41467-024-46426-1 (PMC10959973; doi:10.1038/s41467-024-46426-1)
Supplement: Supplementary file 3 — Description of Additional Supplementary Files [file 41467_2024_46426_MOESM3_ESM.pdf]

DESCRIPTION OF ADDITIONAL SUPPLEMENTARY FILES DOCUMENT

Supplementary Movie 1 : movie-n2.mp4 3D experimental trajectories of 2 zebrafish and the corresponding order parameters.

Supplementary Movie 2 : movie-n3.mp4 3D experimental trajectories of 3 zebrafish and the corresponding order parameters.

Supplementary Movie 3 : movie-n4.mp4 3D experimental trajectories of 4 zebrafish and the corresponding order parameters.

Supplementary Movie 4 : movie-n50.mp4 3D experimental trajectories of 50 zebrafish and the corresponding order parameters.
